# How will second-use of batteries affect stocks and flows in the EU? A model for traction Li-ion batteries

**DOI:** 10.1016/j.resconrec.2019.02.022

**Published:** 2019-06

**Authors:** Silvia Bobba, Fabrice Mathieux, Gian Andrea Blengini

**Affiliations:** a European Commission, Joint Research Centre (JRC), Ispra, Italy; b Politecnico di Torino, Department of Environment, Land and Infrastructure Engineering, Corso Duca degli Abruzzi, 24, 10129, Torino, Italy; c SEIDOR SBS Services, c/Pujades 350, 08019, Barcelona, Spain

**Keywords:** Second-use, Reuse, Europe, Material Flow Analysis (MFA), Materials/energy flows, Li-ion batteries

## Abstract

Although not yet developed in Europe, second-use of traction batteries enables an extension of their lifetime and potentially improves life cycle environmental performance. Li-ion batteries (LIBs) offer the most promising chemistry for traction batteries in electric vehicles (xEVs) and for second-use. Due to the novelty of the topic and the expected increase of e-mobility in the next decades, more efforts to understand the potential consequences of second-use of batteries from different perspectives are needed. This paper develops a dynamic, parameterised Material Flow Analysis (MFA) model to estimate stocks and flows of LIBs after their removal from xEVs along the specific processes of the european value-chain. Direct reuse, second-use and recycling are included in the model and parameters make it customisable and updatable.

Focusing on full and plug-in electric vehicles, LIBs and energy storage capacity flows are estimated. Stocks and flows of two embedded materials relevant for Europe were also assessed (cobalt and lithium). Results showed that second-use corresponds to a better exploitation of LIBs’ storage capacity. Meanwhile, Co and Li in-use stocks are locked in LIBs and their recovery is delayed by second-use; depending on the slower/faster development of second-use, the amount of Co available for recycling in 2030 ranges between 9% and 15% of Co demand and between 7 and 16% for Li. Uncertainty of inputs is addressed through sensitivity analysis.

A variety of actors can use this MFA model to enhance knowledge of second-use of batteries in Europe and to support the effective management of LIBs along their value-chain.

## Highlights

• Second-use entails energy savings but also delays availability of secondary raw materials.
• Different perspectives should be used to assess the effects of second-use of batteries.
• A dynamic and parametrized MFA model describes the value-chain of batteries in Europe.
• Flows of batteries/energy storage capacity/materials are assessed for various scenarios.
• More robust data as model input are needed to proper manage the whole battery system.

## 1 Introduction

E-mobility is key for the decarbonisation of Europe. Sales of electric vehicles (xEVs) are increasing rapidly, both globally and in Europe (Thiel et al., 2016; Zubi et al., 2018). This trend corresponds to an increasing demand for high performance traction batteries for powertrains; this mainly involves Li-ion batteries (LIB) (Lebedeva et al., 2016), which are regarded as the most promising chemistry for xEVs due to their intrinsic characteristics (Blagoeva et al., 2016; IEA, 2018; Pehlken et al., 2017; Schmidt et al., 2016; Zubi et al., 2018). Batteries are recognised as being “at the heart of the industrial revolution” (European Commission, 2018) and the high interest in this technological sector is underlined by both the European Battery Alliance launched at the end of 2017 and the Strategic Action Plan for Batteries (EC, 2018a). However, due to the novelty of the technology and its fast development, more efforts are required to better understand multiple sustainability performances (economic/social/environmental) of LIBs along the whole value-chain (EC, 2018b).

**Fig. 1:**
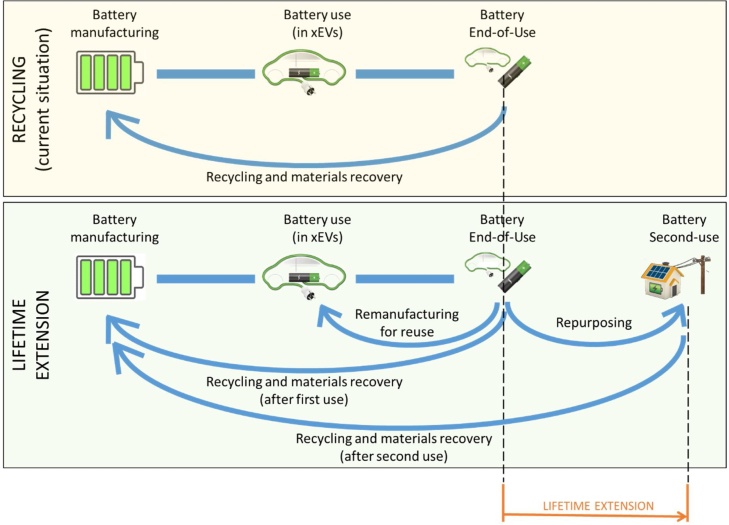
Schematic representation of the end-of-life patterns for LIBs.

The value-chain of batteries in Europe will necessarily need adaptation to the increasing LIB flows along all the life-cycle steps, from their manufacturing to EoL. Concerning manufacturing, “battery production is an imperative for clean energy transition and for the competitiveness of its automotive sector” (EC, 2018a). Moreover, the roadmap of xEV battery technology states that Li-ion based chemistries are likely to dominate the market in the next 30 years (Berckmans et al., 2017; Lebedeva et al., 2016; Zubi et al., 2018), therefore demand and importance of lithium is expected to increase substantially. Among LIBs, prior to 2020, Li-cobalt based chemistries are expected to remain the most important ones for e-mobility, and no substantial changes in chemistries are expected in the European market (Blagoeva et al., 2016; Cusenza et al., 2018; Habib et al., 2017). As a consequence, the demand for raw materials for such batteries will certainly increase (IEA, 2018; Langkau and Tercero Espinoza, 2018). This will include increased demand for some Critical Raw Materials (CRMs) for the EU (EC, 2017a), such as cobalt (Mathieux et al., 2017). The increased production and use of batteries for xEVs will, in time, correspond to an increased in-use stock of raw materials that, with some time delay, will eventually become available for recycling. The fate of xEVs batteries is regulated in the EU by the End-of-Life Vehicles and the Waste Batteries Directives (EU, 2006, 2000), according to which industrial and automotive batteries have to be properly collected and recycled when no longer in service (Fig. 1-top panel). Functional recycling of batteries can produce Secondary Raw Materials (SRMs) that can re-enter the manufacturing process, increasing the degree of circularity and partially avoiding the extraction of raw materials (Mathieux et al., 2017), as emphasised by the European Commission's Circular Economy Action Plan (EC, 2015). At the same time, as is consistent with both the legislative framework (e.g. EU (2008) and the literature (e.g. Tecchio et al. (2017)), the extension of products’ lifetime through their remanufacturing (and consequent reuse) contributes to a more circular economy through the minimisation of wastage and a better resources management. Nevertheless, even though pilot experiences are ongoing (Kampker et al., 2017; Reinhardt et al., 2017), remanufacturing of LIBs and their reuse in xEV is only emerging in Europe, due also to the limited quantity of available batteries (Rohr et al., 2017b).

After their use in xEVs, the residual capacity of traction LIBs ranges between 60% and 80% of the nominal capacity; thus, not surprisingly, there is a high interest and potential for less energy-demanding applications, e.g. residential buildings, uninterruptible power supply, sweepers and driverless transport vehicles (Bobba et al., 2018a; Rehme et al., 2016; Rohr et al., 2017a). In this case, after proper testing and (if needed) repurposing, LIBs can be adopted in second-use applications (Fig. 1-bottom panel). Many examples of second-use of LIBs in various applications are flourishing worldwide, even though they are still at pilot or limited scale (Ahmadi et al., 2014b; Heymans et al., 2014). From an environmental perspective, promising results have already been seen in several R&D activities (Bobba et al., 2018b), especially when second-used batteries are coupled with renewable energy sources (ADEME, 2011; Koch-Ciobotaru et al., 2015; Tamiang and Angka, 2014). Nevertheless, the sustainable development of second-use of LIBs also requires the assessment of aspects not fully covered in the current literature. For instance, estimates of flows of LIBs in Europe would be helpful to understand how collection and transportation schemes should be adapted to the expected volumes and the specific requirements of second-use (e.g. proper handling of LIBs to ease the following second-use), and also the timing of the flow of LIBs sent for recycling.

Although the extension of product lifetime is key to circular economy policies from a materials perspective, longer-lasting LIBs can delay the availability of SRMs from waste recovery (Melin, 2018). The potential growth of second-use is expected to decrease the flows of LIBs available for recycling, and consequently of recoverable materials. On the other hand, second-use could make available an increasing energy storage capacity for applications other than xEV and potentially have positive consequences in terms of energy savings.

The variation of the flows of LIBs in the market and especially the existence of different EoL patterns, i.e. recycling/reuse/second-use, may have consequences that require a comprehensive assessment of the value-chain of LIBs. A more detailed picture of the potential effects of second-use of traction LIBs will help to understand which are the life-cycle steps mainly affected by such a new EoL option. To that end, a comprehensive and flexible model of products and materials flows is needed to show possible interactions and trade-offs between different recovery strategies (i.e. recycling and reuse). This model could also be used to highlight advantages and drawbacks, and finally to identify the best option that should/could be pursued/incentivised in each given context.

An analysis at EU level is needed to fully understand the current and future flow of LIBs in order to obtain suitable information to be used in managing flows of LIBs along their value-chain and to assess the sustainability of a potential business case related to their reuse (EC, 2017b). Similarly, because raw materials are important ingredients for the development of the value-chain (cf. pillar 1 of (EC, 2018c)), the assessment of materials flows along the whole value-chain of LIBs may offer a more complete overview of the LIBs value-chain for stakeholders’ consideration.

### 1.1 Aim and structure of the article

The article presents a flexible and comprehensive material flow analysis (MFA) model developed to assess the variation of the stocks and flows related to LIBs after their use in xEVs in Europe over time, depending on the development of different EoL patterns, with a particular focus on the influence of second-use.

Section 2 gives a concise literature review, highlighting relevant aspects of MFAs of LIBs from available studies, and identifying the relevant knowledge gaps. The proposed MFA model is described in Section 3, highlighting how different EoL options are captured in this model, i.e. both direct reuse of batteries in xEVs, second-use and recycling. Sections 3.1–3.3 respectively describe: i) parameters adopted in the model, ii) the different scenarios and iii) the assessed aspects related to the LIBs value-chain.

This model can be customised to understand the magnitude and the effects of LIBs second-use dynamics (Section 4) compared to other EoL options. To assess the potentialities of the model, the variation of stocks and flows of LIBs in Europe are estimated between 2005 and 2030 in relation to the possible development of second-use. Similarly, the effects of second-use are assessed in terms of energy storage capacity. The variations of the flows of two specific materials (cobalt and lithium) along the LIBs value-chain are quantified for different scenarios. Sections 4.1–4.3 detail the data and assumptions of the analysis, while Section 5 gives the results and the most relevant parameters that emerged from the assessment. This brings us to the discussion (Section 5.3) and conclusions (Section 6).

## 2 Literature review

Table 1 summarises the main outcomes of the literature review in relation to the goal of this study. For each study, the authors focused on the following aspects: temporal and geographical boundaries, type of battery, life-cycle steps reflected in the MFA, assessment of reuse in terms of both remanufacturing and second-use, and the criteria of the MFA analysis (e.g. products, materials).

**Table 1:** Summary of the most relevant aspects for this study available in the scientific literature.

Source		Dynamic/Static MFA	Time frame	Scale of the study			Battery type		"System Boundaries" of the analysis performed in the study				Reuse/remanufacturing (i.e. is reuse/remanufacturing addressed in the study? If yes, how are they considered?)	Criteria of the analysis			
				Global	Regional (region)	National (country)	LIB (type)	Others	Extraction	Manufacturing	Use	EoL		Material	Product	Energy	Other
1	(Simon and Weil, 2013)	S (MEFA)	---	---	---	---	X (NMC, LFP)	---	X	X	---	X	---	Al, Cu, steel, Li, Ni, Co, Mn	X (1 battery)	X	---
2	(Busch et al., 2014)	D	2010 - 2050	---	---	X (UK)	X	---	---	---	X	X	Maximum reuse rate is 95% (highly speculative)	Li, Co, Nd, Pl (fixed breakdown)	EV	---	---
3	(Nakamura et al., 2014)	D	2005 for 100 y	---	---	X (Japan)	---	---	---	X	X	X	---	car steel	---	---	---
4	(Richa et al., 2014)	D (future oriented MFA)	2015 - 2040	---	X (U.S)	---	X (NMC, LFP, LMO, LCO)	---	---	X	X	X	Recycling/Reuse not disaggregated	Al, Co, Cu, Ni, steel, iron, Li, Mn (fixed breakdown of the LIB)	X	---	---
5	(Reuter et al., 2014)	D	2015 - 2050	X	---	---	X (NMC, LFP)	---	X	---	---	X	---	Li, Ni, Mn, Co, iron, natural graphite, phosphate	---	---	---
6	(Schmidt et al., 2016)	S	---	X	---	---	X (NMC, NCA, LCO)	---	X	X	X	---	---	Co, Ni	---	---	---
7	(Pehlken et al., 2017)	D	2014 - 2050	X	---	---	X (NMC, LFP, LMO)	---	---	X	X	X	---	Li, Co (fix breakdown)	---	---	---
8	(Rohr et al., 2017)	D	2015 - 2030	---	---	X (Germany)	X (NMC, LFP, NCA)	---	---	---	---	X	---	Various materials assessed based on their price	X	---	Price
9	(Sun et al., 2017)	D (dynamic trade-linked MFA)	1994 - 2015	X (trade flows)	---	---	X	Li in various products	X	X	(X)	---	---	Li	---	---	---
10	(Olivetti et al., 2017)	forecasts, not a real MFA	2002 - 2025	X (trade flows)	---	---	X (NMC111, NMC622, NMC811, NCA, LCO)	---	X	---	---	---	Reuse discussed qualitatively	Co, Li, Mn, Ni, natural graphite	X	---	---
11	(Ziemann et al., 2018)	D	2010 - 2050	X	---	---	X (NMC, NCA, Li-S)	---	X	X	X	X	---	Li (2010 and 2050)	---	---	---

NMC = lithium-nickel-manganese-cobalt cathode.

NCA = lithium-nickel-cobalt-aluminium cathode.

LFP = lithium-iron-phosphate cathode.

LCO = lithium-cobalt-oxide cathode.

LMO = lithium-manganese-oxide cathode.

Li-S = lithium-sulphur cathode.

The results of the broad-scope review confirmed that only a few studies provide a MFA that considers specific end-of-first-life options of LIBs after their removal from xEVs, including both remanufacturing and second-use. Among these, Busch et al. (2014) adopts highly speculative assumptions (95% of reuse of remanufactured batteries) to prove the potential of the proposed model. In other studies, the MFA estimates the flows potentially available for recycling/reuse without disaggregating the flows of recycling and/or reuse (Richa et al., 2014; Rohr et al., 2017b). Many authors do not consider the option of reuse at all.

The MFA studies analysed are mainly dynamic MFA performed up to 2030 (Rohr et al., 2017b), 2040 (Richa et al., 2014) and 2050 (Busch et al., 2014; Pehlken et al., 2017; Ziemann et al., 2018).

As highlighted by Ardente and Mathieux (2014), geographical and temporal representativeness is relevant for the assessment of various EoL scenarios, e.g. in relation to legislation in force or technological development of a specific area. However, 6 out of 10 studies developed the analysis at global level. Studies at national scale were performed by Busch et al. (2014) and Rohr et al. (2017b), who focused their MFA respectively on UK and Germany, whereas Richa et al. (2014) considers an intermediate scale (U.S.). Since the EU is “*the second largest market of electric vehicles”*, the EU is the geographical boundary of the study performed by Simon et al. (2015); however, no detailed processes of the LIBs value-chain are described in the study. According to the authors’ knowledge, no other dynamic MFA studies including second-use of LIBs has Europe as a geographical boundary. Because the EU level might be the right granularity to address the battery value-chain (cf. battery action plan for manufacturing step or Waste Battery Directive for end-of-life step), it is hence necessary to develop MFA studies for batteries at the EU level.

The relevance of assessing the demand for resources related to the fast increase of LIBs is recognised by the majority of the examined studies. However, data for this assessment are uncertain due to the scarcity of robust data (Olivetti et al., 2017) and the intrinsic level of uncertainty related to the development of new technologies as batteries (Majeau-Bettez et al., 2011; Pehlken et al., 2017). Even though roadmaps of LIBs are available in the literature, changes in LIBs technology and the increase/decrease of materials’ content in the coming decades are considered only by Ziemann et al. (2018) (for Li). Other studies adopt a fixed materials breakdown of LIBs cells for various chemistries.

Finally, the energy storage capacity related to LIBs is usually assessed according to the installed capacity in the xEV market due to xEV demand. However, no details about the potential energy storage capacity of batteries after their use in xEVs is available. Despite the difficulties in estimating the batteries’ lifetime and their residual capacity after they are removed from xEVs (Podias et al., 2018), the estimation of such capacity along the LIBs value-chain could offer a better understanding of the exploitable energy storage capacity.

## 3 The stock and flow model

**Fig. 2:**
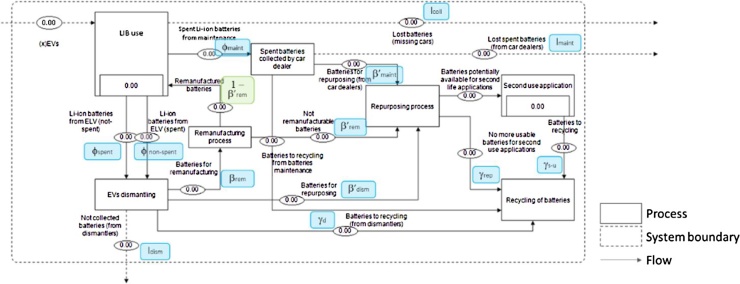
Value-chain model of xEV batteries in Europe according to the stakeholder consultation and the literature review.

For a thorough assessment of the flows of traction LIBs after their use in xEVs, the detailed definition of the processes along the value-chain represents the necessary background of the analysis. Therefore, the fate of products/materials over time could be quantified, as well as product/material losses (Nakamura et al., 2014). The proposed model is a dynamic and parameterised flow and stock model composed of 7 main processes (Fig. 2), as described in this section.

### 3.1 Description of the value-chain of traction batteries in Europe

The life-cycle steps (represented as boxes in Fig. 2) of the LIBs value-chain in Europe (Fig. 2 - dashed box) were identified based on the stakeholders’ interviews during the research. More information about interviews and interviewed stakeholders are available in Bobba et al. (2018b). Literature was used to complement when necessary. Flows of batteries between different processes are represented by arrows.

Traction batteries enter the European market through xEV sales; the first process of the system is therefore their use in xEVs (‘LIB use’), where they are stocked according to their lifetime. After their use, batteries are collected through car dealers (e.g. due to recalls, malfunctions or accidents) (φ_maint_) or dismantlers (when no longer suitable for xEVs) (φ_spent_ and φ_non-spent_) (Timmers, 2016). In the model, batteries from end-of-life vehicles are handled by dismantlers (‘EVs dismantling’), whereas spent batteries substituted during xEV maintenance are handled by car dealers (‘Spent batteries collected by car dealers’). Since not all the batteries are properly removed and collected (Oko Institute, 2016), output flows represent the potential exports from the missing flow of xEV batteries (l_coll_). Other potential losses along the LIBs value-chain are encompassed in the model through l_dism_ and l_maint_.

In Europe, exhausted batteries have to be recycled (EU, 2006, 2000). However, batteries can be removed due to their warranty conditions but still be usable in xEVs (Neubauer et al., 2015b; Willson, 2018). Therefore, before recycling they can be remanufactured and used again in a xEV: in this case, the battery is tested and reconditioned (if necessary) in Europe, and reused again in a xEV (APRA Europe, 2012). This option is considered through the ‘Remanufacturing process’ (β_rem_). According to APRA, remanufacturing of batteries is currently developed in Europe.

After being removed from xEVs, the residual capacity of batteries could potentially be exploited in other applications than xEVs (β’_rem_, β’_dism_, β’_maint_) (Section 1). In the case of second-use, batteries are tested, repurposed (if needed) (‘Repurposing process’) and then used in different applications (‘Second-use application’). This means that a new stock of LIBs within the system should be considered, and the ‘Recycling of batteries’ be delayed in line with the lifetime of LIBs in second-use applications (Rohr et al., 2017b). In line with the goal of the study, and since the landfilling of batteries is banned in Europe (EU, 2006), all batteries in the model are addressed to recycling (either after their first or second life).

### 3.2 Definition of the scenarios for the modelling of flows

The analysis aims to assess the effects of the potential second-use of xEV batteries in Europe, and, in order to test the responsiveness of the model, three different scenarios are considered.

The “Recycling” scenario (‘REP-0’) assumes that, after their removal from xEVs, batteries are collected and addressed to recycling. Considering the current situation in Europe, the market for both remanufacturing and second-use of batteries is not yet developed (Section 1). Therefore, with a view to establishing a *term of reference* scenario, no remanufacturing and no second-use of EV batteries are considered in the ‘REP-0’ scenario.

The ‘REP-0’ scenario is the reference scenario for the comparison with other two scenarios that capture the potential development of a European market for second-use of xEV batteries: “Low second-use scenario” (‘REP-20’) and “High second-use scenario” (‘REP-80’). Bearing in mind the existing barriers/drivers to the development of second-use of xEV batteries (e.g. incentives, legal framework, quantities of LIBs) (Elkind, 2014; Neubauer et al., 2015a; Reinhardt et al., 2017) but also the demonstrated interest in tackling these barriers (e.g. through the Innovation Deal of reuse of xEV batteries^1^), second-use of batteries could gradually develop in the near future. However, due to the novelty of this EoL strategy, the trend of second-use development is unknown and uncertain. In the case of second-using batteries, the batteries’ lifetime is extended in line with both the battery and the application characteristics (Bobba et al., 2018a) and recycling of the battery is consequently postponed in time. The ‘REP-20’ scenario captures the gradual development of LIBs second-use 20% in 2030 (Table 2) through an annual increase of batteries addressed to second-use. Moreover, in line with the current market, and in order to observe the variation of LIB flows related to the arising of second-use, no remanufacturing in Europe is considered in such a scenario.

**Table 2:** Assumptions for the assessed scenarios.

Flow/Process	Parameter	REP-0 SCENARIO	REP-20 SCENARIO	REP-80 SCENARIO
Lost batteries (missing cars)	l _coll_	Annual linear decrease from 40% (in 2005) to 10% (in 2030)	Annual linear decrease from 40% (in 2005) to 10% (in 2030)	Annual linear decrease from 40% (in 2005) to 10% (in 2030)
Remanufacturing	β_rem_	0%	0%	20%
Batteries for repurposing (from dismantlers)	β’_dism_	0%	Annual linear increase from 0% (in 2005) to 20% (in 2030)	70%
Not collected batteries(from dismantlers)	l _dism_	10%	10%	10%
Batteries to recycling (from dismantlers)	γ_dism_	100% - (β_rem_ - β’_dism_ - l _dism_)	100% - (β_rem_ - β’_dism_ - l _dism_)	100% - (β_rem_ - β’_dism_ - l _dism_)
Not remanufacturable batteries	β’_rem_	0%	0%	20%
Batteries for repurposing (from car dealers)	β’_maint_	0%	Annual linear increase from 0% to 20%	100%
Lost spent batteries (from car dealers)	l _maint_	0%	0%	0%
Batteries to recycling (from car dealers)	γ_maint_	100% - (β’_maint_ - l _maint_)	100% - (β’_maint_ - l _maint_)	100% - (β’_maint_ - l _maint_)
Batteries to recycling (from second-use applications)	γ_s-u_	INPUT	INPUT	INPUT
No more usable batteries for second-use applications	γ_rep_	0% from β’_maint_ 0% from β'_rem_ 0% from β’_dism_	0% from β’_maint_ 0% from β'_rem_ 10% from β’_dism_	0% from β’_maint_ 0% from β'_rem_ 10% from β’_dism_

Finally, the ‘REP-80’ scenario was modelled to capture a potential fast development of reuse of batteries, either through remanufacturing or second-use. In their modelling scenarios, Neubauer et al. (2015a) approximate that 80%–90% of batteries will be eligible for repurposing, meaning that “*significant deployments of second use batteries”* will occur after 2030. Natkunarajah et al. (2015) consider that all the batteries could be adopted in second-use applications. Also, the model used by Standridge et al. (2016) envisages that 85% of the batteries will be usable in *post-vehicle-applications*. In conclusion, based on the literature and also in line with the goal of the paper, the ‘REP-80’ scenario considers that 20% of the non-spent LIBs will be remanufactured (i.e. used again in xEVs), and the majority of the removed LIBs will be adopted in various applications other than in xEVs.

**Fig. 3:**
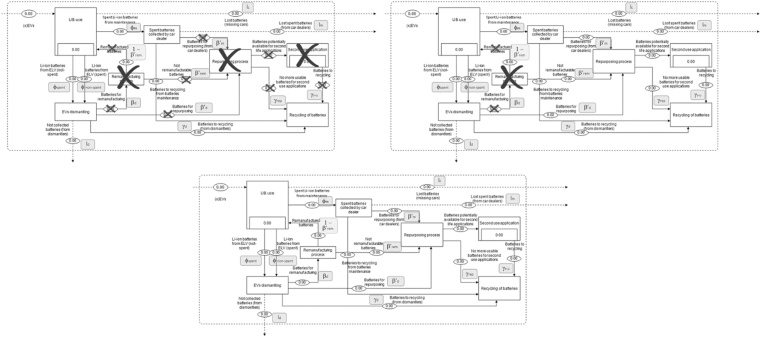
Differences in the value-chain processes in Europe in line with the assessed scenario. Black crosses highlight processes with no flows of batteries.

Fig. 3 gives an overview of the main differences between flows assessed in the three scenarios illustrated above.

### 3.3 Assessed aspects addressed by the stocks and flows model

The proposed model allows the assessment of the variation of xEV battery flows along the various processes of the value-chain in line with the input data. Furthermore, it is constructed in such a way that it enables to consider different aspects related to traction batteries, such as materials embedded in LIBs and/or energy capacity.

Throughout their lifetime, LIBs provide energy to xEVs but potentially also to other applications. The fact that a battery’s capacity decreases during its lifetime depending on the battery’s characteristics and use is one of the most relevant parameter to be considered for second-use applications (Podias et al., 2018; Rohr et al., 2017a). Considering the capacity of different types of batteries, the model can be used to estimate the flows of energy storage capacity associated with a battery’s flows at different steps of its life.

Finally, the model can also be used to assess the stocks and flows of specific materials embedded in LIBs along their value-chain. Despite the intrinsic uncertainty related to new technologies (Pehlken et al., 2017), the model enables to estimate the flows of materials relevant for Europe, for instance cobalt or lithium embedded in specific LIBs chemistries.

## 4 Application of the model to traction LIBs in Europe

The MFA model introduced in Section 3.1 is applied to the xEVs LIBs in Europe between 2005 and 2030, in particular to those used for both plug-in and full xEVs (i.e. PHEVs and BEVs). Hybrid electric vehicle (HEVs) LIBs were excluded mainly due to their characteristics: in HEVs, the conventional combustion engine is the main power source (electricity is generated on board) (EUROBAT, 2014; Huss et al., 2013; McEachern, 2012) and the level of electrification of HEV batteries is lower than for traction batteries used in BEVs and PHEVs. Consequently, also according with (EUROBAT, 2015), second-use of LIBs is considered only for PHEVs and BEVs.

Input data used for the modelling are illustrated in Section 4.1, with a detail on energy storage capacity (Section 4.2) and embedded materials (Section 4.3).

### 4.1 Data and assumptions to model the stocks and flows of traction LIBs

Consistent with the MFA methodology, the law of conservation of matter is used to establish the metric calculation and the relationships between the processes of the system (Brunner and Rechberger, 2004; Müller et al., 2014). STAN software^2^ is used to estimate the stocks and flows of the system (Fig. 2) for all the assessed scenarios.

**Fig. 4:**
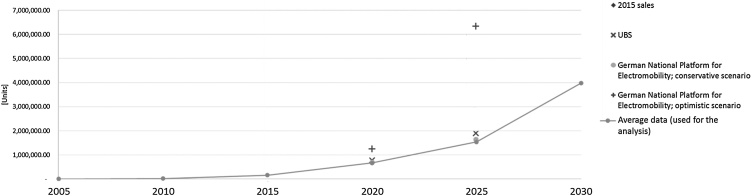
Projected sales of new PHEV and BEV vehicles in Europe for 2015–2025 (Lebedeva et al., 2016) (dots) and average of the collected data for this analysis (line).

The estimate of BEVs and PHEVs sales in Europe between 2005 and 2030 is based on several sources available from the literature, e.g. Bank of America Merrill Lynch (2016); Blagoeva et al. (2016); EAFO (2016); Kampman et al. (2011). The authors’ own calculations were necessary since often data are aggregated or provided at global level. Excluding peaks of sales mainly related to optimistic scenarios (e.g. in Kampman et al.(2011)), the elaboration of projected European sales confirmed the trend illustrated by Lebedeva et al. (2016) (Fig. 4). In the model, the time interval considered for the analysis is 1 year.

The penetration rate of LIBs in the automotive sector is expected to increase from 15% to 90% between 2010 and 2025 (Pillot, 2014). Moreover, based on Chmura (2016), traction batteries for PHEV are only LIBs and for BEVs are predominantly LIBs. For the analysis, a penetration rate for LIB in electric vehicles of 70%, 80% and 100% (linear increasing) is assumed respectively for 2005, 2010 and after 2015.

The average lifetime of a vehicle is about 10 years (EUROSTAT, 2018a), even though various aspects contribute to make this aspect highly uncertain and this value varies across different studies (Richa et al., 2014; Sweeting and Winfield, 2012). Battery lifetime depends on several factors, e.g. driving style and frequency of charging (Daimler, 2015; Podias et al., 2018). Due to the lack of specific data about battery lifetime, in the literature lifetime is considered as ranging between 5 and 15 years (Ahmadi et al., 2014a; Neubauer et al., 2015b; Sathre et al., 2015; Wang et al., 2014), even though it is expected to increase up to 15 years in 2030 (EUROBAT, 2015). Such estimates are also aligned with manufacturers’ warranties (e.g. Leaf battery (Cobb, 2014)). Traction LIBs are removed from xEVs for different reasons, e.g. low capacity (spent battery), end of the warranty/leasing period, accidents. To capture this variability and to better reflect the reality (e.g. possible early replacements, EoL users’ behaviours), the discrete lifetime distribution proposed by Richa et al. (2014) is assumed: 10% of batteries have a lifetime of 6 years, 40% of batteries have a lifetime of 8 years, 40% of batteries have a lifetime of 10 years, 10% of batteries have a lifetime exceeding 12 years. The uncertainty of such aspects would require a more in-depth analysis and real data to estimate the real lifetime of LIBs in both first and second life (Podias et al., 2018).

The collection rate of both automotive and industrial batteries in Europe is nearly 100% (EC, 2014; Mudgal et al., 2014). Nonetheless, about 30% of the vehicle waste flow (including batteries) in the EU is unknown whereabouts (Oko Institute, 2016) and, according to the consulted stakeholders, the abovementioned collection rate is overestimated. Due to the lack of data on collection of traction LIBs (Stahl et al., 2018), an initially conservative collection rate is assumed for 2005 (60%) and then it is assumed to rise constantly to 90% in 2030. It is also assumed that batteries collected by car dealers have already reached their EoL, so that they are no longer usable in xEVs; in this case, the analysis entails the substitution of the battery only if the car still has more than 2 years’ lifetime.

Batteries potentially adoptable in second-use applications should be tested to assess their conditions (e.g. state-of-health) and the best suitable application (Koch-Ciobotaru et al., 2015; Rehme et al., 2016). Defining the lifetime of batteries in such applications is challenging since it depends on both the battery’s and the systems’ characteristics; also, a lack of data is often addressed through estimates or average data (Bobba et al., 2018a, 2018b). Based on an average value of 88 years, this aspect was varied in the sensitivity analysis in order to assess its relevance to the overall results (Section 5.2).

Table 2 summarises the main assumptions and the main differences between the three scenarios illustrated above.

### 4.2 Data and assumptions used to model the stocks and flows of energy storage capacity

Battery capacity (and its consequent lifetime) is an important limiting factor for the development of xEVs, and continuous efforts by the automotive and batteries industries are tending to increase it (Bank of America Merrill Lynch, 2016; EEA, 2016; Ziemann et al., 2018; Zubi et al., 2018). Due to confidentiality issues, few data about the forecasted capacity of traction batteries are available in the literature. In contrast to Simon and Weil (2013), in which fixed values are adopted to assess the flows of energy storage capacity in the near future, several sources were used to estimate the evolution of LIBs capacity over time (Table 4).

It is assumed that the capacity of LIBs when reaching their EoL (i.e. ‘φ_m_’ and ‘φ_spent_’) is 60% of the nominal capacity of the battery, whereas for other batteries (i.e. ‘φ_non-spent_’) it is 80%. Due to the uncertainty of this aspect (Section 4.1), a sensitivity analysis is performed and illustrated in Section 5.2.

### 4.3 Data and assumptions used to model the stocks and flows of embedded materials

As illustrated in Section 1, Li-Co based chemistries will remain the most promising chemistry for e-mobility before 2020. As a consequence of their increasing demand, demand of both Li and Co is also expected to increase substantially (IEA, 2018; Langkau and Tercero Espinoza, 2018).

Among the Li-Co chemistries, the NMC (nickel-manganese-cobalt) and NCA (nickel-cobalt-aluminium) are the most widely adopted for BEVs and PHEVs due to their suitable characteristics (e.g. energy density and durability) and the forecasted decrease of costs (Zubi et al., 2018). To assess the potentiality of the model in estimating the stocks and flows of materials embedded in LIBs, the analysis focuses on Co and Li embedded in these two chemistries. Due to the lack of data about the market share of such chemistry up to 2030, several sources were used to gather information (Blagoeva et al., 2016; JRC, 2013; Pillot, 2017). Results of elaborations and average shares are summarised in Table 3.

**Table 3:** Market share of NMC and NCA batteries included in the analysis.

	NMC 111	NMC 532	NMC 622	NMC 811	TOT NMC	NCA
2005	30.00%	0.00%	0.00%	0.00%	30.00%	8.00%
2010	12.00%	18.00%	0.00%	0.00%	30.00%	10.00%
2015	12.04%	16.17%	5.16%	1.03%	34.41%	11.55%
2020	23.95%	20.96%	11.97%	2.99%	59.87%	10.97%
2025	15.33%	21.46%	18.40%	6.13%	61.32%	9.90%
2030	9.00%	27.00%	36.00%	18.00%	90.00%	10.00%
Note that in 2030 all the LIB market is assumed to be made of NMC and NCA chemistries

**Table 4:** Summary of the data used for both the energy flows and the material content flows analysis.

		Residual capacity [kWh/battery]	Cobalt content [kg/battery]		Lithium content [kg/battery]	
			NMC 111	NCA	NMC	NMC
PHEV	2005	6.23	2.38	1.44	0.79	1.25
	2010	6.23	3.38	1.55	0.79	1.25
	2015	8.10	4.38	1.65	2.01	2.03
	2020	10.11	5.75	2.50	2.49	2.09
	2025	11.23	6.56	2.88	3.19	2.67
	2030	12.98	6.56	2.88	3.88	3.26
BEV	2005	17.58	14.34	8.44	4.64	6.23
	2010	17.58	14.04	8.13	4.64	6.23
	2015	28.75	13.74	7.81	5.49	6.09
	2020	38.70	20.98	9.12	7.43	7.62
	2025	39.65	20.83	9.14	8.27	8.48
	2030	45.20	20.83	9.14	8.86	9.08

* for the calculations, the Co percentages in the cathode are: 18.24% for NMC532, 12.16% for NMC622 and 6.06% for NMC811.

Concerning materials content, also because the cost of Co supply heavily affects the price of battery packs, its proportion in LIBs is expected to decrease after 2025 in different chemistries. For instance, new chemistries with lower Co content are available already, e.g. NMC 523, 622, and 811 instead of NMC 111 (IEA, 2018; Perks, 2016; Pillot, 2017); also, the use of composite cathodes is another strategy to decrease the Co content (Cusenza et al., 2018; Patry et al., 2014). Mainly due to the lack of data and the uncertainty of sources, steady values concerning materials content are usually used to assess the materials flow (Section 2). However, to quantify the flows of specific materials along the various processes of the value-chain, technology development should be considered. Also in this case, several sources were consulted (Blagoeva et al., 2016; Gruber et al., 2011; JRC, 2013; Petersen, 2018; Tivander, 2016; Ziemann et al., 2018) and Table 4 depicts the analysis inputs.

## 5 Results and discussion

The data and information presented in Section 4.1 were used as the input to model the ‘REP-0’, the ‘REP-20’ and the ‘REP-80’ scenarios through the STAN software. This section reports the main outcomes of the analysis.

### 5.1 Results of the stocks and flows analysis

**Fig. 5:**
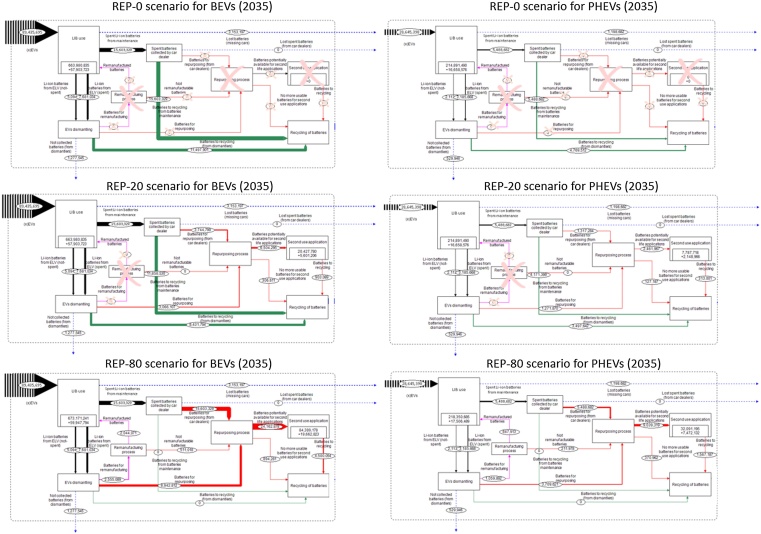
Energy capacity storage of LIBs in BEVs (left) and PHEVs (right) in 2035 in Europe for different scenarios.

In general, the gradual increase of xEVs sales in Europe will not significantly affect the materials and capacity flows before 2025. Then, major differences will concern the recycling flows of LIBs after their removal from xEVs (green arrows in Fig. 5) and the second-used LIBs (red flows in Fig. 5) (for interpretation of the references to colour in this figure legend, the reader is referred to the web version of this article).

**Fig. 6:**
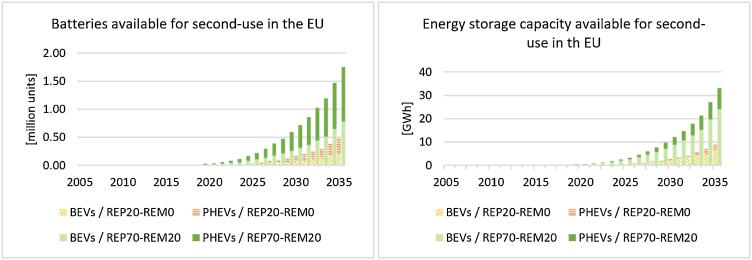
Batteries available for second-use applications in Europe (left) and the respective energy storage capacity (right). The ‘REP-0’ scenario is not reported since no second-use occurs.

With a gradual increase of repurposing of xEVs batteries (‘REP-20’ scenario), in 2025 more than 38,500 LIBs could be adopted in second-use applications in Europe, of which 53% will be from PHEVs and 47% from BEVs (Fig. 6). Results show that this amount of batteries corresponds to a residual capacity of 0.6 GWh: in turn this corresponds to about 14% of the energy storage capacity for self-consumption applications in Europe (Kessels et al., 2017). Even though the amount of BEVs and PHEVs batteries available for second-use is similar (about 70,500 and 91,500 respectively in 2030), about 73% of the abovementioned capacity is provided by batteries used in BEVs, which are characterised by higher energy density than batteries used in PHEVs. Focusing on the ‘REP-80’ scenario, the amount and the capacity of batteries available for second-use is 4 times higher in comparison to the ‘REP-20’ scenario (Fig. 6).

In the case of a gradual development of second-use, LIBs addressed to recycling in 2030 and 2035 are estimated to be respectively 1.23 and 1.25 times lower than those in the ‘REP-0’ scenario, where no second-use occurs. In this case, through the model, it is possible to estimate the energy storage capacity of non-exploited LIBs due to direct recycling rather than second-use: in 2030 it is about 13.5 GW h for the ‘REP-0’ scenario, almost 11 GW h for the ‘REP-20’ scenario and almost 2 GW h for the ‘REP-80’ scenario (73% from BEVs’ LIBs).

The delay in terms of available LIBs entering the recycling process can be estimated for the different scenarios: in 2020, about 40,500 LIBs are addressed to recycling in the ‘REP-0’ scenario. The same amount will be recycled with a delay of half a year in the ‘REP-20’ scenario and of 7 years for the ‘REP-80’ scenario.

**Fig. 7:**
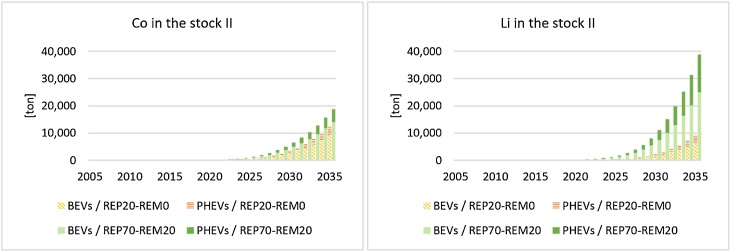
Cobalt (left) and Lithium (right) stocked in second-use applications in Europe. The ‘REP-0’ scenario is not reported since no second-use occurs.

Second-use of LIBs results in the creation of new stock. Looking at materials embedded in LIBs, it is possible to estimate the amount of Co and Li stocked in second-use applications and consequently the time shift before they are addressed to recycling. Focusing on the ‘REP-20’ scenario, in 2030 about 3400 tonnes of Co will be stocked in xEV LIBs adopted in second-use applications (74% of which will be embedded in BEVs’ LIBs). This amount is almost 2 times higher in the ‘REP-80’ scenario. This means that the Co available for recycling in 2030 is 19% lower in the ‘REP-20’ scenario than the ‘REP-0’ scenario. Similarly for Li, in 2030 the stock of Li in second-use batteries will be about 2200 tonnes (67% of which in BEVs’ LIBs) in the ‘REP-20’ scenario and about 11,000 tonnes in ‘REP-80’ scenarios (Fig. 7).

**Fig. 8:**
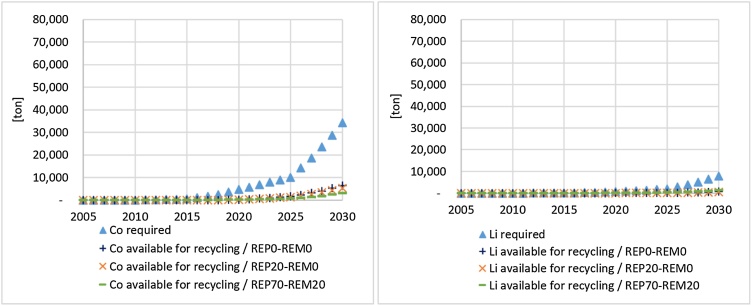
Cobalt (left) and Lithium (right) in LIBs and available for recycling in Europe.

To have a more complete overview of the amount of materials entering the system through LIBs and the materials available for recycling, these flows are illustrated in Fig. 8. Assuming that all the Co and Li could be used for LIB manufacturing, results show that the delay of Co and Li available for recycling caused by the second-use of LIBs does not significantly decrease the materials required for LIB manufacturing. For instance, in 2030 the Co entering the recycling process through LIBs ranges between 3000 tonnes (‘REP-80’) and 6,500 tonnes (‘REP-0’) whereas the Co entering the EU embedded in LIBs is greater than 34,000 tonnes. Moreover, it is worth noting that the quantity of SRMs should consider the efficiency of the recycling processes according to the technology applied. Currently, 94% of the input Co can be recovered, whereas Li recovery requires more complex treatment and its recovery is still not available in Europe at industrial scale (Lebedeva et al., 2016; Mathieux et al., 2017).

### 5.2 Sensitivity analysis

Data and information about the lifetime of LIBs in both first and second life is lacking (see Section 2). Moreover, next generations of LIBs will be more performant, in order to meet consumers’ expectations, so that a higher LIB capacity should be expected. These values are varied through a one-at-time variation (Igos et al., 2018) and details of the performed analysis are given in the supplementary materials. The main outcomes are discussed below.

Concerning the lifetime of LIBs in second-use applications, upper and lower values of lifetime are considered for the sensitivity analysis. According to the literature (Bobba et al., 2018a,b), these values are respectively 5 and 12 years. The results of this variation are given in Figures S1, S2, S3 and S4. Focusing on the ‘REP-20’ scenario, if LIBs in second-use applications last 5 years rather than 12 years, the embedded Co sent for recycling is around 200 tonnes higher. This difference grows to more than 1000 tonnes in 2035. A similar trend is observed for Li.

To assess the relevance of the residual capacity of LIBs when removed from xEVs, an “early replacement” and a “late replacement” are considered. For the “early replacement” it is assumed that the non-spent batteries (i.e. φ_non-spent_) are collected when their residual capacity is 90% of the nominal capacity, whereas the spent batteries (i.e. φ_maint_ and φ_spent_) are collected at 70% of the nominal capacity. For the “late replacement”, the non-spent batteries (i.e. φ_spent_) are collected when their residual capacity is 70% of the nominal capacity, whereas the spent batteries (i.e. φ_maint_ and φ_non-spent_) are collected at 60% of the nominal capacity. Compared to the “late replacement”, the “early replacement” results in 2.14 GW h more to be potentially used in second-use applications in 2030 (increasing to 5.88 GW h in 2035) (Figure S3). Therefore, the “early replacement” could be an interesting option for utilities especially where high volumes of LIBs are adopted in second-use applications (‘REP-80’).

### 5.3 Discussion of results

The stock and flows model proposed in this paper describes in detail the value-chain of batteries after their removal from xEVs in Europe. In the model, all the possible EoL patterns (i.e. direct reuse, second-use and recycling) are captured and modelled through the adoption of different parameters, which is a new approach when compared to the available studies in the literature (see Section 2). Although second-use of LIBs is not fully developed in Europe, it seems to be a promising option with a view to decreasing the costs of LIBs (and xEVs) and also to improve the sustainability of LIBs (Ambrose et al., 2014; Bobba et al., 2018a; Kirmas and Madlener, 2017). However, most of the available studies address the variation of stocks and flows of LIBs (or specific materials embedded in LIBs), providing an overview of the available batteries entering in the EoL flows (e.g. Richa et al. (2014)), but they do not specifically focus on the effects caused by the variation of flows in line with the extension of the LIBs’ lifetime (Pehlken et al., 2017; Ziemann et al., 2018).

The expected increase of batteries is also related to the increase in energy storage capacity available on the market and the increase in raw materials required for their manufacture: these include some critical raw materials for the EU (e.g. cobalt); all these aspects are relevant when assessing the sustainability of a new EoL option in a complex system. Analysed MFA studies focus on specific aspects related to LIBs, e.g. LIB flows (e.g. Busch et al. (2014); Richa et al. (2014)) or embedded materials (e.g. Busch et al. (2014); Pehlken et al. (2017); Richa et al.(2014); Ziemann et al. (2018)), without combining them.

Different scenarios (including new ‘REP-x’ scenarios) and the consequences of the development of various EoL patterns in Europe could be assessed, highlighting relevant aspects related to both direct reuse and second-use of recycling (e.g. higher amount of LIBs available for recycling and consequently more SRMs) vs lifetime extension of LIBs with a consequent delay in recycling but a better exploitation of their storage capacity. Where second-use gradually increases (‘REP-20’ scenario), analysis results show that if LIBs are second-used, almost 3 GW h of energy storage capacity deriving from their residual capacity can be used in 2030, for example in residential buildings. This means that, especially if batteries are coupled with renewable energies, the share of renewable energy in Europe can be increased and the second-use of traction LIBs can potentially avoid the production of fresh/new storage batteries (Viswanathan and Kintner-Meyer, 2011).

The processes along the battery value-chain in Europe will need to be adapted to the increasing flows of LIBs in the coming decades, and a more in-depth knowledge of LIBs/capacity/materials flows may be helpful for the various actors involved in battery management along the whole value-chain. The knowledge of the flows of batteries (in terms of both units and storage capacity) may be useful both for collectors, e.g. to better organise collection schemes (Bobba et al., 2018b), and also utilities to estimate the overall capacity of LIBs that, after proper testing and (if needed) repurposing can be potentially exploited in various applications. Depending on the quantity of batteries available in the future and the development of their performance (Rohr et al., 2017b), the creation of a business case related to second-use is also an opportunity for car manufacturers.

In Europe, the relevant Co and Li in-use stocks are locked in LIBs, but their demand is expected to increase significantly, as highlighted by the assessment of the future demand of such materials, e.g. in Busch et al. (2014); Pehlken et al. (2017); Sun et al. (2017); Ziemann et al. (2018). In particular, the demand in Co for NMC and NCA chemistries in Europe in 2020 is estimated to be 4650 tonnes, increasing to 10,000 tonnes in 2025 and 34,200 in 2030. In case of second-using LIBs, batteries available for recycling, and consequently available SRMs, will be postponed in time. Considering current recycling rates of materials from LIBs, the amount of LIBs addressed to recycling subsequently will give recyclers an overview of the quantity of SRMs recoverable from LIBs flows in the event of the slower/faster development of second-use. At the same time, the creation of a stock related to the second-used LIB may support the development of more specialised and efficient recycling processes in the future, with larger volumes involved, a higher recovery rate of specific materials and better quality of SRMs, which may be relevant for the recovery of materials such as lithium. Results of the performed analysis show that, if second-use is the main EoL option in Europe (‘REP-80’ scenario), in 2030 about 3000 tonnes of Co embedded in NMC and NCA chemistries will be addressed to recycling. This value is higher in case of the slower development of second-use in Europe (5000 tonnes)^3^ . Such low values could even be a concern, since this kind of recycling rate is generally used to monitor progress towards a circular economy (see for example indicator 7a of the Circular Economy Monitoring Framework - (EC, 2018b; EUROSTAT, 2018b). Considering that second-use offers some relevant circularity opportunities, this means that novel indicators of re-use would need to be proposed for monitoring purposes.

Because of the data gaps, mainly related to the novelty and the fast development of battery technology, input data are quite uncertain and difficult to obtain; often fixed data or assumptions or aggregated data are used in MFAs (e.g. in Busch et al. (2014)). The performed analysis varied two parameters highly affected by uncertainty:: lifetime in second-use applications and the residual capacity of LIBs when removed from xEVs. The longer/shorter lifetime of batteries could affect the flows of batteries, energy storage and materials within the assessed system. Lifetime is recognised as a very important parameter for the development of e-mobility. However, due to a lack of information, real data about the lifetime of LIBs in both xEVs and other applications are needed (Podias et al., 2018). In this study, the lifetime of LIBs in xEVs is assumed to have a discrete distribution, aligned with Richa et al. (2014), whereas a fixed value is used for lifetime in second-use applications; this parameter was then varied in order to assess its influence on the final results. In addition, the potential early/late removal of LIBs from xEVs is assessed through the variation of the residual capacity of LIBs. It is observed that no major variations occur when varying the second life of LIBs, whereas the variation of the residual capacity could have a more relevant impact in terms of energy storage capacity, especially for the early replacement of LIBs. Due to the uncertainty of such parameters, a more in-depth analysis of the adopted parameters within the analysis is recommended in further work.

Overall, the proposed model can be used to extrapolate information on flows of LIBs, energy capacity storage and embedded materials within Europe. In line with the goal of the analysis and the life-cycle steps of interest, users can extract different type of information (e.g. quantities of specific materials recoverable through recycling, capacity potentially exploitable in second-use applications, losses of batteries along the value-chain, etc.). Different scenarios (including new ‘REP-x’ scenarios) and the consequences of the development of various EoL patterns can be assessed, highlighting relevant aspects related to both direct reuse and second-use of recycling (e.g. higher amount of LIBs available for recycling and consequently more SRMs) vs lifetime extension of LIBs with a consequent delay in recycling but a better exploitation of the storage capacity of LIBs. Moreover, the detailed description of the value-chain of LIBs represents an added value for the monitoring of specific flows of LIBs/storage/materials along the different processes in the life-cycle.

## 6 Conclusions

A dynamic stock and flows model was developed to describe the life-cycle steps and processes along the value-chain of Li-ion batteries (LIBs) after their removal from electric vehicles in Europe. All the possible end-of-life (EoL) patterns (i.e. direct reuse, second-use and recycling) are captured in the model, even though second-use of traction LIBs is not yet developed in Europe. Parameters make the model flexible and customisable according to the available input data; furthermore, different scenarios can help identify circular economy aspects and highlight the effects of different EoL options under different aspects on stocks and flows of LIBs.

Focusing on LIBs removed from both BEVs and PHEVs, stocks and flows of LIBs in Europe are quantified along the value-chain between 2005 and 2035 through 3 different scenarios: second-use will not occur in Europe (‘REP-0’ scenario), second-use will progressively develop in Europe (‘REP-20’ scenario) and second-use will become the main EoL option in Europe (‘REP-80’ scenario). The variation of stocks and flows of both LIBs and their energy capacity storage along the life-cycle steps were assessed for all scenarios. Furthermore, the assessment is also enlarged to estimate the materials flows of two materials embedded in LIBs for which a high interest is confirmed by both the literature review and policy documents: cobalt and lithium.

Results pointed out that second-use allows a better exploitation of storage capacity of LIBs. On the other hand, recovery of cobalt and lithium to be recirculated in the European economy is delayed due to lifetime extension of LIBs. The relevance of this delay also depends on the development and deployment of recycling capacities at full-scale: the current high recycling rate of cobalt may contribute to a decrease in the demand for primary cobalt for LIBs (about 3000 tonnes of cobalt to be sent for recycling in 2030 in the ‘REP-80’ scenario); concerning lithium, its potential recirculation cannot decrease the demand for lithium for LIBs as it is not currently recovered at industrial scale. Lack of robust data inevitably affects the uncertainty of results; further data collection/elaboration efforts (e.g. sales of xEVs, Weibull distribution to model the lifetime of batteries) and sensitivity analyses addressing relevant parameters are recommended for future analyses. Moreover, further scenarios related to possible policy interventions (e.g. bans of some specific LIBLIB chemistry or substance, minimum recycling content, re-use targets) could also be analysed so that the model supports decision making.

The novelty of the second-use of LIBs triggers complex changes that require more-in depth assessment in order to capture the effects and trade-offs of the potential extension of battery lifetime in various applications and to support the proper management of the whole system with awareness of the peculiarities of the specific life-cycle processes along the LIBs value-chain. Combined with an environmental assessment (Bobba et al., 2018a), this paper contributes to a more-in depth knowledge of the second-use of batteries and its potential effects in Europe. More work on economic and also social aspects (e.g. creation of new jobs, decrease of battery costs to increase affordability, etc.) should be done to provide a comprehensive overview of the development of the assessed system.

## Disclaimer

The views expressed in the article are personal and do not necessarily reflect an official position of the European Commission.

## Footnotes

1 “The Innovation Deal focuses on propulsion batteries and will assess whether existing EU legal provisions and the transposition to national or regional law hamper the use of batteries in a second-life application or otherwise discriminate any technology that might be necessary for second-life applications” (EC, 2017c).

2 http://www.stan2web.net/.

3 This is about 9% and 15% of the Co entering the European market through xEV sales for the “REP-80” and “REP-20” scenarios respectively.
