# Supplementary material for: How will second-use of batteries affect stocks and flows in the EU? A model for traction Li-ion batteries
Source: Resour Conserv Recycl. 2019 Jun;145:279–91. doi: 10.1016/j.resconrec.2019.02.022 (PMC7099760; doi:10.1016/j.resconrec.2019.02.022)
Supplement: Supplementary file 1 [file mmc1.docx]

How will second-use of batteries affect stocks and flows in the EU? A model for traction Li-ion batteries

Silvia Bobba ^1,2^, Fabrice Mathieux ^1*^, Gian Andrea Blengini ^1,2^

^1^ European Commission, Joint Research Centre (JRC), Ispra, Italy

^2^ Politecnico di Torino, Department of Environment, Land and Infrastructure Engineering, Corso Duca degli Abruzzi, 24 – 10129 Torino, Italy

^*^ F. Mathieux (corresponding author): [fabrice.mathieux@ec.europa.eu](mailto:fabrice.mathieux@ec.europa.eu)

# Supplementary materials

**Sensitivity analysis (see section 5.2)**

The lifetime of LIBs in second-use applications varied between a lower (5 years) and an upper value (12 years). Figure S1 and S2 show the variation of the Co and Li stocked in second-use application when battery lifetime is varied, whereas Figures S3 and S4 show the variation of the available Co and Li for recycling when battery lifetime is varied.
In both cases, the higher variation refers to the amount of Co and Li stocked in second-use applications. For Co (Figure S1), the Co stocked in second-use application in 2030 in the longer lifetime (i.e. 12 years) is about 500 tonnes higher than in the shorter lifetime (i.e. 5 years). For Li (Figure S2), this difference is about 350 tonnes.
Focusing on the available materials available for recycling, results show that the variation of the lifetime in second-use applications does not significantly affect the flows of materials entering in the recycling process. However, in terms of absolute values, the difference between the Co available for recycling (Figure S3) in the longer second-life is about 260 lower than in the shorter second-life. This difference is about 180 tonnes for Li (Figure S4).

**
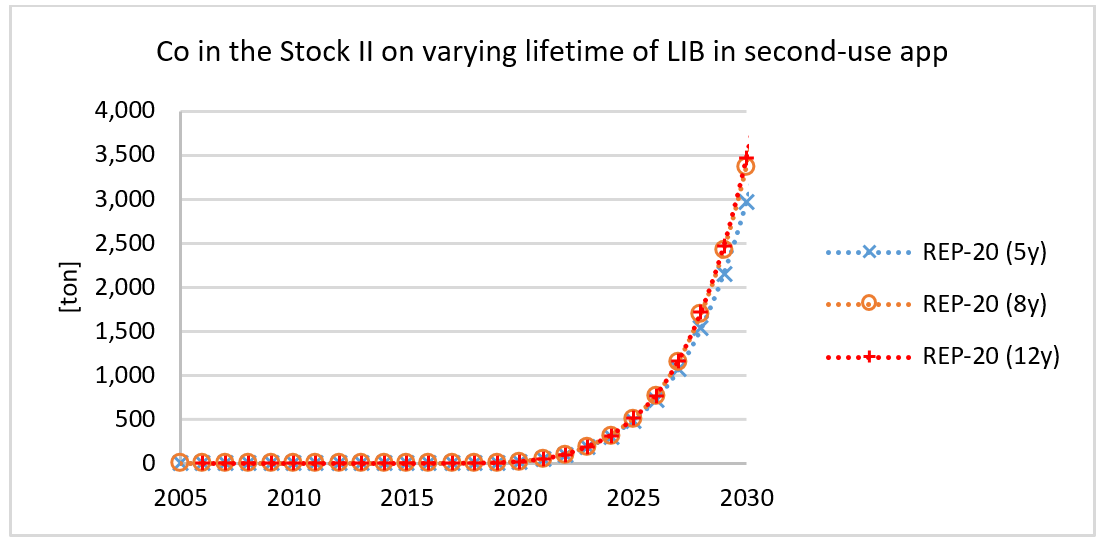
**

**Figure S1**: Variation of the Co stocked in second-use applications in Europe with the variation of the lifetime of batteries during their second-use


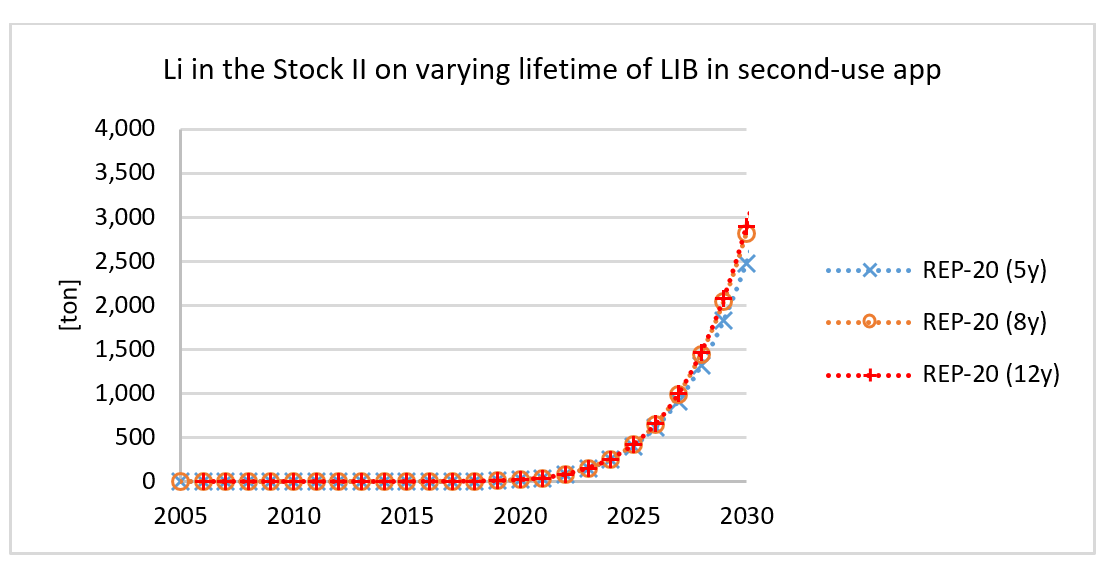


**Figure S2**: Variation of the Li stocked in second-use applications in Europe with the variation of the lifetime of batteries during their second-use


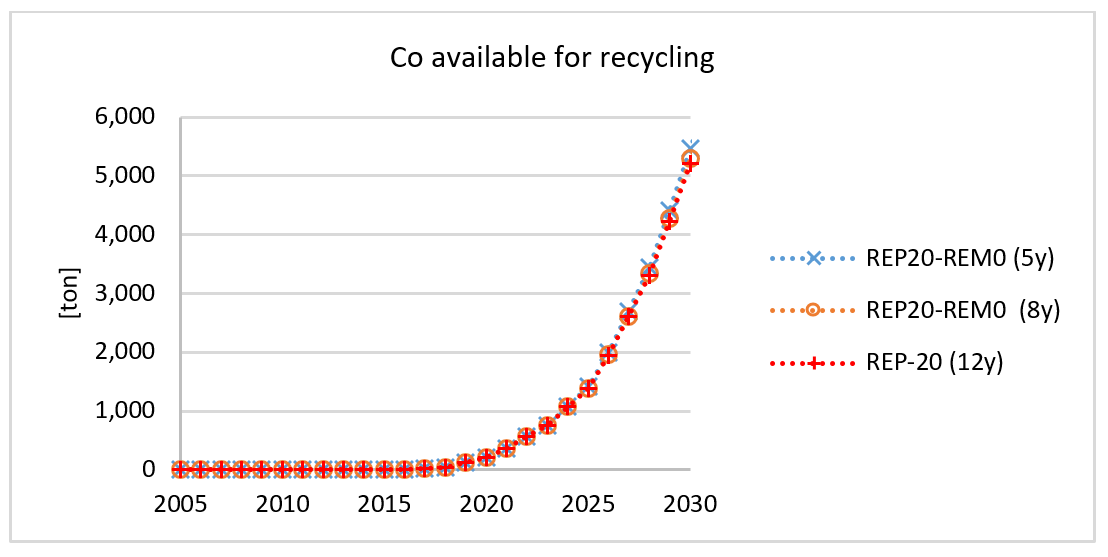


**Figure S3**: Variation of the Co available for recycling with the variation of the lifetime batteries during their second-use

**
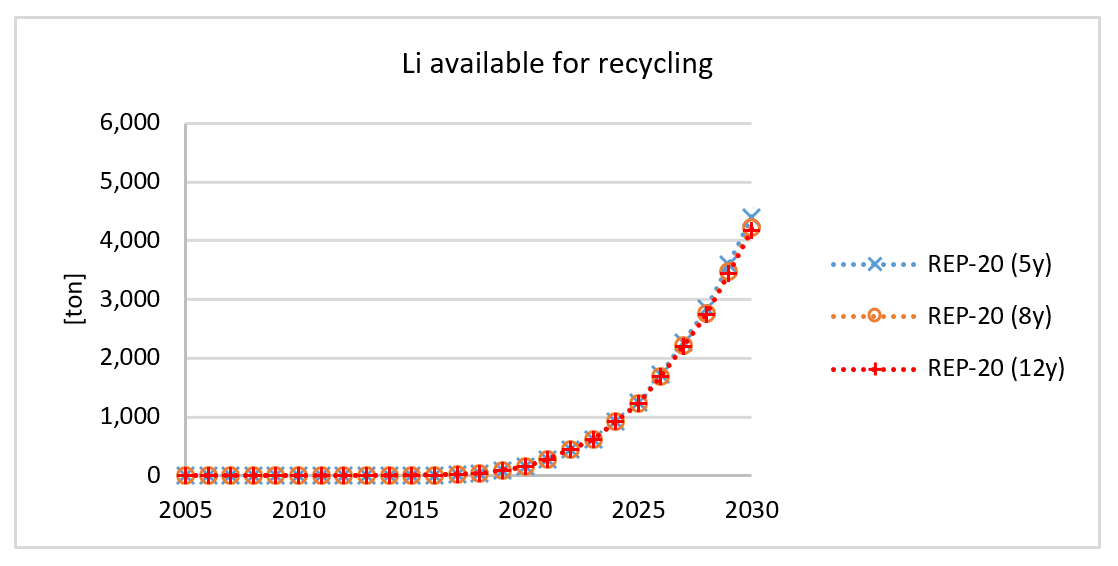
**

**Figure S4**: Variation of the Li available for recycling with the variation of the lifetime batteries during their second-use

In order to assess the relevance of the residual capacity of LIBs when removed from xEVs, the early and late replacement options are considered. Figure S5 shows that the variation of the energy storage capacity potentially available for second-use applications due to early replacement creates a significant increase only in the ‘REP-80 scenario’, which means that early replacement entails important energy savings compared to late replacement (+ 6 GWh) if second-use represents the main EoL option for LIBs.


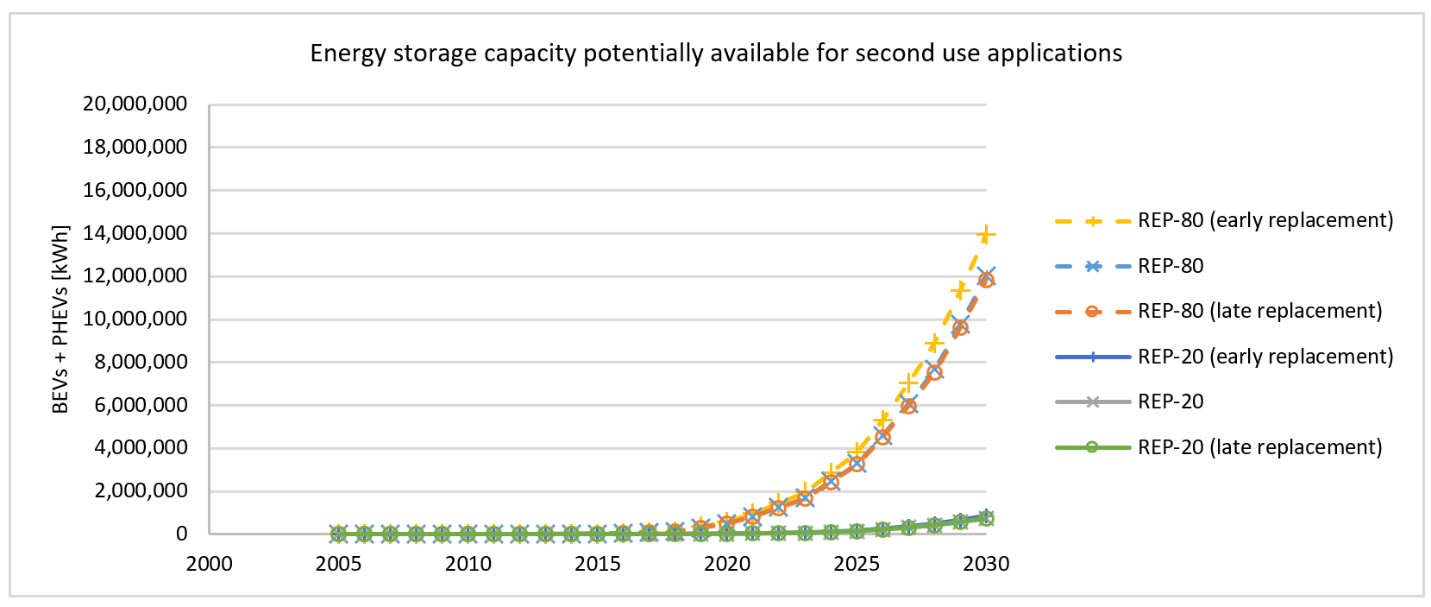


**Figure S5**: Variation of the energy storage capacity potentially available for second use applications with the variation of the lifetime of the second-used batteries
